# Author Correction: Potential impact and cost-effectiveness of long-acting injectable lenacapavir plus cabotegravir as HIV treatment in Africa

**DOI:** 10.1038/s41467-026-72202-4

**Published:** 2026-04-17

**Authors:** Andrew Phillips, Jennifer Smith, Loveleen Bansi-Matharu, Kenly Sikwese, Cissy Kityo, Charles Flexner, Marco Vitoria, Nathan Ford, Meg Doherty, Zack Panos, David Ripin, Matthew Hickey, Diane Havlir, Monica Gandhi, Michael Reid, Paul Revill

**Affiliations:** 1 Institute for Global Health, University College London, London, UK; 2 AfroCAB, Lusaka, Zambia; 3 Joint Clinical Research Centre, Kampala, Uganda; 4 Johns Hopkins University School of Medicine, Baltimore, MD USA; 5 Department of Global HIV, Hepatitis and Sexually Transmitted Infections Programmes, WHO, Geneva, Switzerland; 6 Children’s Investment Fund Foundation, London, UK; 7 Clinton Health Access Initiative, New York, NY USA; 8 University of California San Francisco, San Francisco, CA USA; 9 US Department of State’s Bureau of Global Health Security and Diplomacy – United States President’s Emergency Plan for AIDS Relief (PEPFAR), Washington DC, USA; 10 University of York, York, UK

Correction to: *Nature Communications* 10.1038/s41467-025-60752-y, published online 1 July 2025

In the version of this article initially published, there was an error in the values shown in Table 3 for people with HIV who have ever taken lenacapavir + cabotegravir, where the percentage of people with virologic failure was originally listed as “7% (1% − 29%) 10% (9% − 10%)”; the table is now corrected to list “0% (0% − 0%) 0% (0% − 0%)” in the HTML and PDF versions of the article.

